# ALICE: Conceptual Development of a Lower Limb Exoskeleton Robot Driven by an On-Board Musculoskeletal Simulator

**DOI:** 10.3390/s20030789

**Published:** 2020-01-31

**Authors:** Manuel Cardona, Cecilia E. García Cena, Fernando Serrano, Roque Saltaren

**Affiliations:** 1 Centre for Automation and Robotics (CAR), Universidad Politécnica de Madrid (UPM), 28006 Madrid, Spain; cecilia.garcia@upm.es (C.E.G.C.); roquejacinto.saltaren@upm.es (R.S.); 2 Faculty of Engineering, Universidad Don Bosco (UDB), San Salvador, El Salvador; 3 Faculty of Engineering and Architecture, Universidad Tecnológica Centroamericana (UNITEC), Frente a Residencial, V-782 Boulevard Kennedy, Tegucigalpa, Honduras; serranofer@eclipso.eu

**Keywords:** adaptive control, exoskeleton robot, muscle driven simulator, quaternions, rehabilitation

## Abstract

Objective: In this article, we present the conceptual development of a robotics platform, called ALICE (Assistive Lower Limb Controlled Exoskeleton), for kinetic and kinematic gait characterization. The ALICE platform includes a robotics wearable exoskeleton and an on-board muscle driven simulator to estimate the user’s kinetic parameters. Background: Even when the kinematics patterns of the human gait are well studied and reported in the literature, there exists a considerable intra-subject variability in the kinetics of the movements. ALICE aims to be an advanced mechanical sensor that allows us to compute real-time information of both kinetic and kinematic data, opening up a new personalized rehabilitation concept. Methodology: We developed a full muscle driven simulator in an open source environment and validated it with real gait data obtained from patients diagnosed with multiple sclerosis. After that, we designed, modeled, and controlled a 6 DoF lower limb exoskeleton with inertial measurement units and a position/velocity sensor in each actuator. Significance: This novel concept aims to become a tool for improving the diagnosis of pathological gait and to design personalized robotics rehabilitation therapies. Conclusion: ALICE is the first robotics platform automatically adapted to the kinetic and kinematic gait parameters of each patient.

## 1. Introduction

Robotics exoskeletons are a technology in continuous development mainly in the medical and military fields. Considering the medical domain, both upper and lower limb exoskeletons obey two paradigms: rehabilitation and augmentation. There is a clear conceptual difference between them. The rehabilitation exoskeletons are designed to follow a specific therapy/treatment. It is assumed that patients are going to enhance their mobility in the rehabilitation therapy. This robot must be useful for a wide range of patients considering weight, height, gender, and disease, among others. Moreover, mechanical parameters, on-board sensors, and the software and hardware architecture are expected to be flexible enough in order to adapt the device to each particular patient.

However, in an augmentation robotics exoskeleton, it is assumed that patients are not going to improve their mobility after undergoing therapy because there is a permanent degree of disability. Such cases need a personalized device that enhances the patient’s abilities and improves his/her quality of life as much as possible.

In this last case, we have an additional consideration. Patients diagnosed with a degenerative disease such as multiple sclerosis have also cognitive impairment, then it is necessary to endow robots with enough knowledge to automatically adapt to each situation.

In the last two decades, the interest in this particular application of robots has been increasing because it arises as a cost/effective solution for the increasing demand of the traditional rehabilitation system. Rehabilitation therapy with robots ensures a greater number of repetitive movements than manual rehabilitation. As a consequence, the natural proprioceptive input coming from limb movements stimulates the brain and spinal cord neuroplasticity, which is the key for restoring the mobility of the affected limb(s) in cases of some neuromuscular disorders [1].

An example is the well known application ALEXproject from Delaware University with at least three versions of lower limb exoskeletons [2,3,4]. All versions are focused on kinematic control of the gait, where joint forces, as well as visual and audio signals are fed back to the control loop in order to minimize the errors in a trajectory. Other examples with the same concept are LOPES [5,6,7] or MLLRE [8]. The reader is refereed to the literature for a deep review of mobile and stationary lower limb exoskeletons [9,10,11,12,13,14].

In rehabilitation, the main goal is to restore the movements as much as possible. In contrast, in augmentation, the goal is to delay the onset of the most severe symptoms. However, in both cases, kinematic and kinetic parameters must be considered in the robot control loop. Most advanced control strategies consider the patients’ internal muscle forces measured by electromyography sensors. Others prototypes also include electrical nerve signals from the spine or limbs to generate appropriate activation signals for actuators. Finally, the last generation of robotic exoskeletons are those that use directly the brain signal to activate the robot using the potential movement intention.

In this paper, we present the conceptual design of a robotic exoskeleton platform, ALICE, with both capabilities: rehabilitation and augmentation. Kinetic and kinematic data are measured or estimated for both the patient and robot. These data are fed back to the control loop in order to restore or maintain a particular movement. By merging these data into a real-time adaptive PDcontrol architecture, we ensure automatically adapting the therapy to the personal condition of the patients. The robot’s kinematic data are measured by its internal sensors, while the kinematic data of patients’ limb are measured by inertial unit sensors placed on the limb. Kinetic data are computed or simulated: we use the dynamical model of the robot to compute the joints/torque forces in order to control its trajectory while the patient’s kinetic data are estimated by a validated full bio-mechanical simulator running on-board [15]. This novel concept turns ALICE into a sensor–motor robotic exoskeleton suitable for personalized gait analysis. Moreover, it can be used as an augmentation robot for permanently disabled people or patients with multiple sclerosis disease to contribute with the torques required when the patient loses his/her mobility.

**Figure 1:**
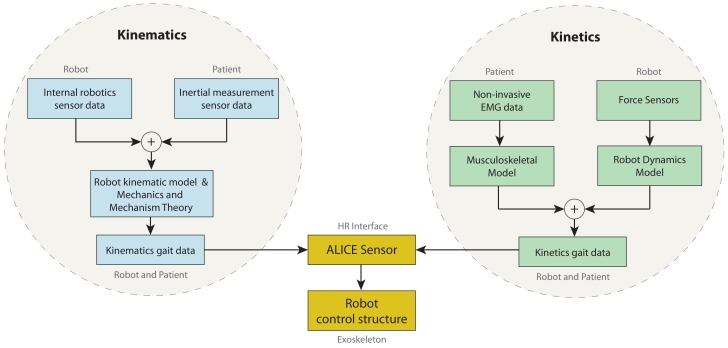
ALICE concept.

Figure 1 shows the ALICE concept. Because an exoskeleton is a wearable device, it could be assumed that the kinematic data of the patient’s gait are computed through the internal sensors of the robots in addition to the inertial unit sensors placed on the patient’s limbs. However, this hypothesis is not valid for kinetics data. The robot’s forces are computed by its dynamical model, while the gait’s kinetic data are computed by the musculoskeletal model using the information given by external EMG sensors. The capabilities included in this platform turns ALICE into the first robotics device able to be used for diagnosis. At the same time, this robotics mechanism can be used to perform repetitive rehabilitation tasks; therefore, data (measured and/or estimated) are the feedback in the control architecture.

This article is organized as follow. In Section 2, the ALICE project is presented. In Section 3, the kinematics and dynamics modeling is described. In Section 4, the gait kinetics is analyzed, and the musculoskeletal model, as well as, the gait capture wireless system are presented. Section 5 presents the control strategy proposed based on a PD adaptive controller that includes the kinetic data of the patient.

## 2. Brief Description of the ALICE Project

Over the last 15 years, research in the field of rehabilitation robotics has grown exponentially, as shown in [16,17,18]. The mismatch between increasing demand of rehabilitation services and the available resources to cover it is one of the leading causes. As a consequence, rehabilitation sessions’ length reduces from typically 45 min to 30 min, as the wait list exceeds the recommended time. Moreover, most part of rehabilitation therapies are carried out “one-by-one”, and the workload on physiotherapists is very high. Physiotherapists cannot meet the demand and, in some cases, end up becoming patients due to the physical overload to which they are exposed [19].

The main goal of the ALICE project is to provide rehabilitation medical staff with a tool that helps in the rehabilitation process while simultaneously reducing their workload. At the same time, ALICE is an advanced sensor that provides all necessary information about the patient’s movements and improves the diagnosis and prognosis of a particular injury. ALICE is a three link lower limb rehabilitation exoskeleton (hip, femur, and tibia) and includes three electric actuators. The first version of ALICE included four degrees of freedom (DoF) and three active actions (flexion/extension of the hip, abduction/adduction of the hip, and flexion/extension of the knee). The dorsal/plantar flexion of the ankle is a passive DoF, and it could be considered as the limitation of this study. Therefore, the range of usability of this device is restricted to those injuries where active action in this joint is not required.

### Mechanical Design

From the mechanical point of view, ALICE is adjustable for adult patients with femur and tibia lengths between 35 and 50 cm and a pelvic width from 29 to 40 cm. In addition, it is designed to assist in the gait pattern’s diagnosis, as well as to measure the patient’s recovery in the gait rehabilitation objectively. Furthermore, ALICE could be used in patients who have suffered some neuro-muscle disorders with the limitation stated before.

Currently, the project is in the process of validating components/subsystems and performing laboratory tests and simulations in a real environment. ALICE has an average level of technological maturity (TRL) (level of technological preparation, NASA) of 5.

The major drawbacks to extend the use of this advanced technology lie in (a) economic aspects, as robotic exoskeletons are still expensive, (b) the credibility and acceptability gap in both rehabilitation staff and patients, and (c) the regulatory requirements because being a diagnosis and a rehabilitation machine, it is forced to undergo the necessary clinical trials. Every step further in the TRL scale of ALICE’s project keeps in mind these three key points.

**Figure 2:**
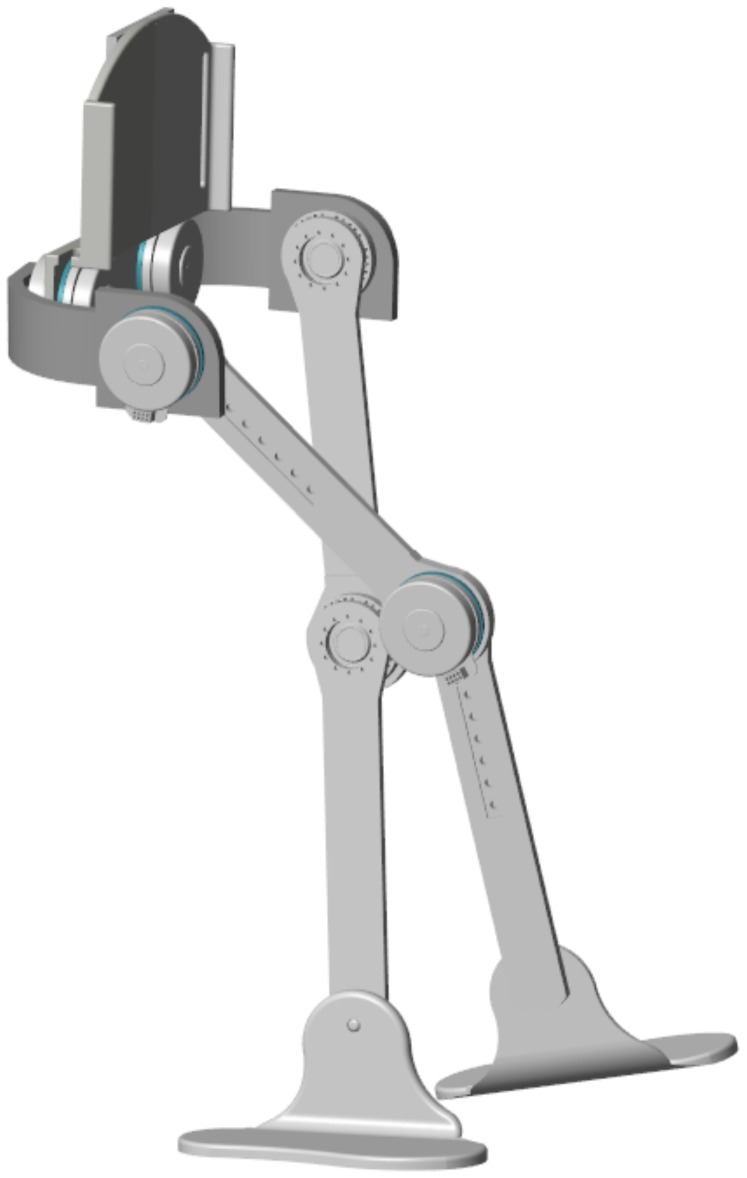
CAD of the exoskeleton robot for rehabilitation, ALICE.

ALICE has a support that can be adhered to the pelvis and two adjustable elements that adapt to the leg and tibia, respectively, as shown in Figure 2.

For each lower limb member, there is a mechanism with three active joints and one passive joint. The first active joint corresponds to flexion/extension of the hip, the second active joint to abduction/adduction of the hip, and the third active joint to flexion/extension of the knee. Finally, the degree of passive freedom corresponds to the ankle that allows dorsal and plantar flexion.

The range of mobility of each joint is restricted to the values found in the literature [20,21,22], and are shown in Table 1. Thus, the workspace and dexterity of the mechanism is strongly constrained by the range of work of the joints.

**Figure 3:**
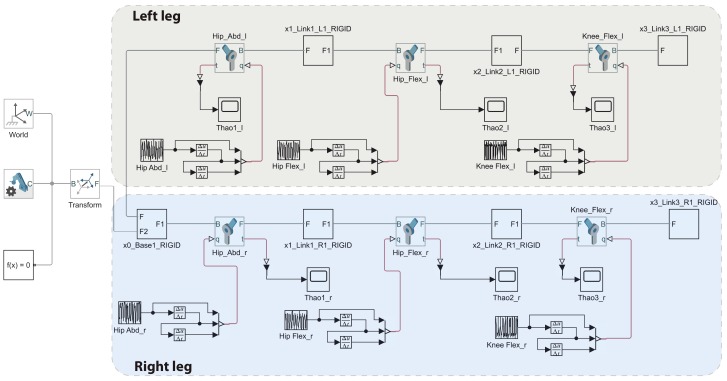
ALICE, Simulink^®^, Simscape Multibody^TM^ equivalent model.

In order to select the actuators, patient’s weight was added to the CAD model (considering a maximum weight of 135 kg) and then exported to Simulink^®^. The equivalent Simulink^®^ model allows evaluating the needed torques for a pure movement and integrating all the MATLAB^®^ and Simulink^®^ features. Figure 3 depicts the equivalent Simulink^®^ model.

**Figure 4:**
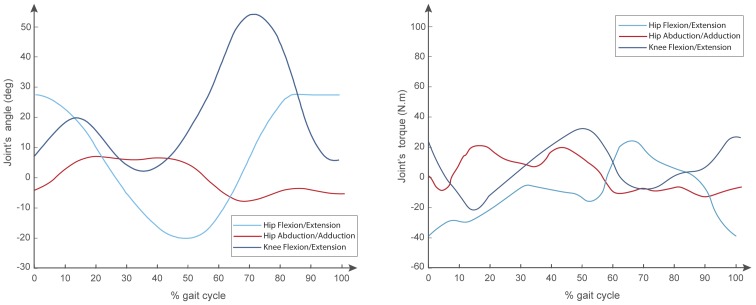
Joint’s angles for a gait cycle and the joint’s required torque.

Once the model was exported, activation signals for normal gait were set as reference joint’s angles, and the required torque was determined. Figure 4 shows the joint’s angles of reference and the torque predicted for this reference value.

Figure 4 shows that the maximum torque required for hip flexion/extension, hip abduction/adduction, and knee flexion/extension for a gait cycle was around 38, 21 and 32 N·m, respectively. Moreover, for pure movements, the flexion/extension, hip abduction/adduction, and knee flexion/extension for a gait cycle were around 42, 23 and 20 N·m, respectively.

For actuators’ selection, the catalogs from Maxon motors and Harmonic Drive were considered because these catalogs show a wide variety of combinations of their products, i.e., motors, gear boxes, drivers, and encoders integrated. According to the maximum values obtained in the simulation, the suitable motors are the Maxon EC Flat 90 series and the Harmonic Drive Cobalt Line 17, 100 CPM, obtaining an average torque of 51 N·m. Furthermore, the control board recommended is the EPOS2 70/10 digital position controller.

## 3. Kinematics and Dynamics Modeling

**Figure 5:**
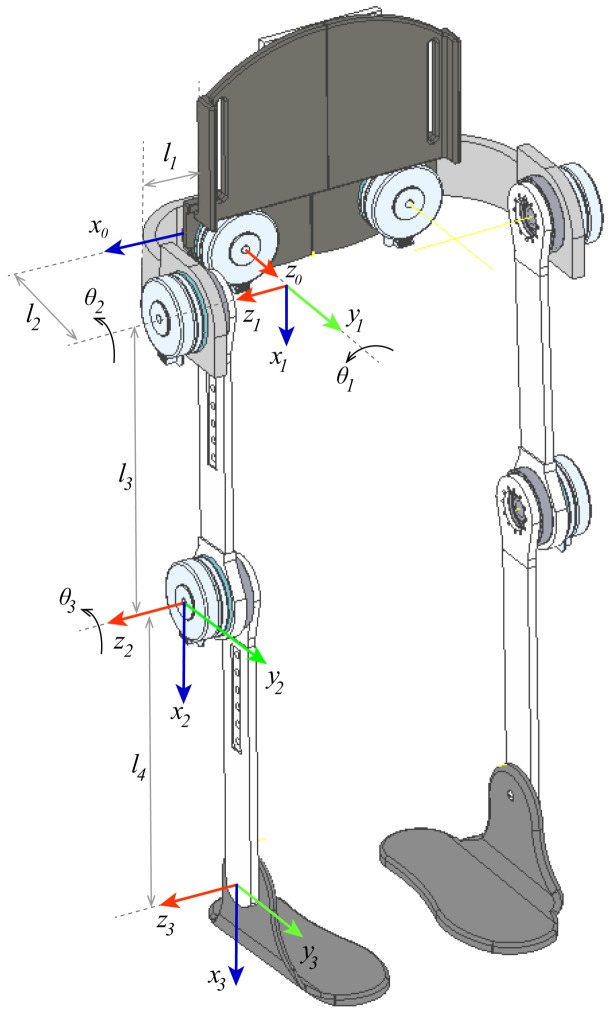
Reference frames using Denavit–Hartenberg (DH) representation.

For the kinematic analysis, the unit quaternions’ theory was used due to its simple and compact mathematical representation compared to other methods, such as the Denavit–Hartenberg (DH) method [23,24,25]. However, starting from the reference frames defined by the DH methods and then applying on the transformation matrix the properties and operation defined for the unit quaternions, it is possible to get linear and angular velocities straightforwardly (see Figure 5).

### 3.1. Transformation Matrices

Let us consider the following unit quaternion:

(1) $$b=b_{0}+b_{1}i+b_{2}j+b_{3}k$$

The transformation matrix is given by:

(2) $$T_{i}^{i-1}=\left[\begin{array}{cc} R_{i}^{i-1} & P_{i}^{i-1}\\ 0 & 1 \end{array}\right]$$

where $R_{i}^{i-1}\in {ℜ}^{3\times 3}$ is the rotation matrix and $P_{i}^{i-1}$ is the translation vector. The rotation matrix in terms of unit quaternions can be obtained by rotating an angle $\theta_{i}$ around the *z* axis and an angle $\alpha_{i}$ around the *x* axis.

Thus, defining the vector $z_{i}$:

(3) $$z_{i}=cos\left(\theta_{i}\right)+sin\left(\theta_{i}\right).k$$

The rotation around *z* will be given by:

(4) $${}_{z}R_{i}^{i-1}=\left[z_{i}⊗d_{x},z_{i}⊗d_{y},d_{z}\right]$$

where $d_{x}=i$, $d_{y}=j$ and $d_{z}=k$. Moreover, a rotation of an angle $\alpha_{i}$ around the *x* axis yields,

(5) $$x_{i}=cos\left(\alpha_{i}\right)+sin\left(\alpha_{i}\right).i$$

Therefore, the rotation around *x* will be given by:

(6) $${}_{x}R_{i}^{i-1}=\left[d_{x},x_{i}⊗d_{y},x_{i}⊗d_{z}\right]$$

On the other hand, the translations matrices in the *z* and *x* axes are given by:

(7) $${}_{z}P_{i}^{i-1}=\left[\begin{array}{c} 0\\ 0\\ s_{i} \end{array}\right]{\;}_{x}P_{i}^{i-1}=\left[\begin{array}{c} a_{i}\\ 0\\ 0 \end{array}\right]$$

The rotation and translation matrices are used to define the global transformation matrix necessary to obtain the kinematics of the exoskeleton following the DH method.

Furthermore, the translations in the *z* and *x* matrices are given in (8) and the transformation matrices in (9):

(8) $$S_{i}^{i-1}=\left[\begin{array}{cc} I & {}_{z}P_{i}^{i-1}\\ 0 & 1 \end{array}\right]\;A_{i}^{i-1}=\left[\begin{array}{cc} I & {}_{x}P_{i}^{i-1}\\ 0 & 1 \end{array}\right]$$

(9) $$T_{i}^{i-1}=\left[\begin{array}{cc} {}_{z}R_{i}^{i-1} & 0\\ 0 & 1 \end{array}\right]S_{i}^{i-1}A_{i}^{i-1}\left[\begin{array}{cc} {}_{x}R_{i}^{i-1} & 0\\ 0 & 1 \end{array}\right]$$

where *I* is an identity matrix. Therefore, the transformation matrix of the exoskeleton is given by:

(10) $$T_{3}^{0}=T_{1}^{0}T_{2}^{1}T_{3}^{2}$$

### 3.2. Speed Matrices

In order to obtain the angular velocities, it is necessary to define both the linear and angular coordinates as expressed in [26]. Consider the following linear coordinate vector $X={\left[x,y,z\right]}^{T}$ and the linear velocities with respect to the origin:

(11) $${\dot{X}}_{1}^{0}={\dot{P}}_{1}^{0}\;{\dot{X}}_{2}^{0}={\dot{P}}_{2}^{0}\;{\dot{X}}_{3}^{0}={\dot{P}}_{3}^{0}$$

Furthermore, consider the angles of the exoskeleton joints $q={\left[q_{1},q_{2},q_{3}\right]}^{T}$ on the *z* axis with the rotation angles $\Phi ={\left[\theta ,\phi ,\psi \right]}^{T}$. Therefore, the angular velocities with respect to the base axis are given by [26]:

(12) $$\begin{array}{ccc} \omega_{1}^{0} & = & {\dot{\Phi }}_{1}^{0}={\dot{q}}_{1}k\\ \omega_{2}^{0} & = & {\dot{\Phi }}_{2}^{0}={\dot{q}}_{1}k+{\dot{q}}_{2}R_{1}^{0}k\\ \omega_{3}^{0} & = & {\dot{\Phi }}_{3}^{0}={\dot{q}}_{1}k+{\dot{q}}_{2}R_{1}^{0}k+{\dot{q}}_{3}R_{2}^{0}k \end{array}$$

Defining the vector $k={\left[0,0,1\right]}^{T}$, the expressions given in (12) can be written in matrix form:

(13) $$W_{1}=\left[k,0,0\right]\;W_{2}=\left[k,R_{1}^{0}k,0\right]\;W_{3}=\left[k,R_{1}^{0}k,R_{2}^{0}k\right]$$

Finally, the $W_{i}$ matrices for i=1,2,3 give an important relationship since the velocity in all axes can be obtained from the angular velocities of each actuated joint:

(14) $$\omega_{i}^{0}=W_{i}\dot{q}$$

### 3.3. Dynamic Modeling

To obtain the exoskeleton dynamics, the Euler–Lagrange method is implemented by first defining the Lagrangian as shown below,

(15) $$L=\sum_{i=1}^{3}K_{i}-\sum_{i=1}^{3}P_{i}$$

where $K_{i}$ is the kinetic energy and $P_{i}$ is the potential energy for links i=1,2,3. In (16), each term of the previous equation is defined.

(16) $$\begin{array}{ccc} K & = & \frac{1}{2}\sum_{i=1}^{3}{\dot{X}}_{i}^{T}M_{i}{\dot{X}}_{i}+\frac{1}{2}\sum_{i=1}^{3}\omega_{i}^{T}I_{i}\omega_{i}\\ P & = & \sum_{i=1}^{3}m_{i}gX_{i}^{T}D_{y} \end{array}$$

where $D_{y}={\left[0,1,0\right]}^{T}$, $X_{i}$, and $\omega_{i}$ are defined in (11) and (12), and the $M_{i}$ matrix is given by,

(17) $$M_{i}=\left[\begin{array}{ccc} m_{i} & 0 & 0\\ 0 & m_{i} & 0\\ 0 & 0 & 0 \end{array}\right]$$

where $m_{i}$ is the mass for link i=1,2,3, $I_{i}\in {ℜ}^{3}$ is the inertia matrix for links i=1,2,3, and *g* is the gravity constant. Making a change of variables, the Lagrangian becomes $L=\frac{1}{2}{\dot{\bar{X}}}^{T}M\dot{\bar{X}}+\frac{1}{2}\Omega^{T}I\Omega -{\bar{X}}^{T}D_{y}^{\prime }$, where,

(18) $$\begin{array}{c} \bar{X}={\left[X_{1},X_{2},X_{3}\right]}^{T}\;\Omega ={\left[\omega_{1},\omega_{2},\omega_{3}\right]}^{T}\\ \Omega ={\left[{\dot{\Phi }}_{1},{\dot{\Phi }}_{2},{\dot{\Phi }}_{3}\right]}^{T}\;\Phi ={\left[\Phi_{1},\Phi_{2},\Phi_{3}\right]}^{T} \end{array}$$

and:

(19) $$\begin{array}{c} M=\left[\begin{array}{ccc} M_{1} & 0 & 0\\ 0 & M_{2} & 0\\ 0 & 0 & M_{3} \end{array}\right]\;I=\left[\begin{array}{ccc} I_{1} & 0 & 0\\ 0 & I_{2} & 0\\ 0 & 0 & I_{3} \end{array}\right]\\ D_{y}^{\prime }=\left[\begin{array}{c} D_{y}m_{1}g\\ D_{y}m_{2}g\\ D_{y}m_{3}g \end{array}\right] \end{array}$$

Next, making the following change of variables:

(20) $$\begin{array}{c} Q={\left[{\bar{X}}^{T},\Phi^{T}\right]}^{T}\;\dot{Q}={\left[{\dot{\bar{X}}}^{T},{\dot{\Phi }}^{T}\right]}^{T}={\left[{\dot{\bar{X}}}^{T},\Omega^{T}\right]}^{T}\\ \ddot{Q}={\left[\ddot{\bar{X}},{\ddot{\Phi }}^{T}\right]}^{T}={\left[{\ddot{\bar{X}}}^{T},{\dot{\Omega }}^{T}\right]}^{T} \end{array}$$

we have:

(21) $$L=\frac{1}{2}{\dot{Q}}^{T}\left[\begin{array}{cc} M & 0\\ 0 & I \end{array}\right]\dot{Q}-Q^{T}\left[\begin{array}{c} D_{y}^{\prime }\\ 0 \end{array}\right]$$

Finally, applying the Euler–Lagrange equation given by,

(22) $$\frac{d}{dt}\left[\frac{\partial L}{\partial \dot{Q}}\right]-\frac{\partial L}{\partial Q}=\tau_{Q}$$

where $\tau_{Q}={\left[0_{3},\tau_{\Phi }^{T}\right]}^{T}$. The exoskeleton dynamics equation is:

(23) $$\left[\begin{array}{cc} M & 0\\ 0 & I \end{array}\right]\left[\begin{array}{c} \ddot{\bar{X}}\\ \dot{\Omega } \end{array}\right]+D_{Q}=\left[\begin{array}{c} 0\\ \tau_{\Phi } \end{array}\right]$$

where $D_{Q}={\left[D_{y}^{T^{\prime }},0\right]}^{T}$.

Using the virtual torque $\tau_{qn}$, it is possible to define a transformation $\tau_{\Phi n}$ to implement the input of each exoskeleton actuator. Thus, let us consider the following equation,

(24) $$\begin{array}{c} I_{n}{\dot{\omega }}_{n}=\tau_{\Phi n}\\ I_{n}\left[{\dot{W}}_{n}\dot{q}+W_{n}\ddot{q}\right]=\tau_{\Phi n} \end{array}$$

$\dot{\omega }$ can be obtained from the first derivative of (14); thus, the expression given by (24) can be rewritten as,

(25) $$D_{n}\ddot{q}+C_{n}\dot{q}=\tau_{\Phi n}$$

where $D_{n}=I_{n}W_{n}$ and $C_{n}=I_{n}{\dot{W}}_{n}$. Multiplying both sides of (25) by $D_{n}^{-1}$ yields:

(26) $$\ddot{q}+D_{n}^{-1}C_{n}\dot{q}=D_{n}^{-1}\tau_{\Phi n}$$

Furthermore, to transform the forces and moments of the exoskeleton links to the actuator torques for n=3, the $\tau_{\Phi n}\to \tau_{qn}$ transformation given by $\tau_{qn}=D_{n}^{-1}\tau_{\Phi n}$ is required.

## 4. Kinetic Gait Modeling

A full musculoskeletal model for muscle driven simulation was included in our platform to compute internal muscle force data from patients. The model was created using an open source software called MSMS and exported to the MATLAB environment, which is based on the well known biomechanical model of Hill–Zajac [27,28,29]. In spite of this, the insertion of each bone, muscle, tendon, and ligaments was done manually by the authors. Fourteen bones, 88 Hill-type muscle–tendon segments, 10 ligament segments for each knee, and 6 joints for each lower limb were modeled [15]. The muscle–tendon parameters were based on those reported by Cardona et al. [15], Arnold et al. [22], Ward at al. [30], and Rajagopal at al. [31].

**Figure 6:**
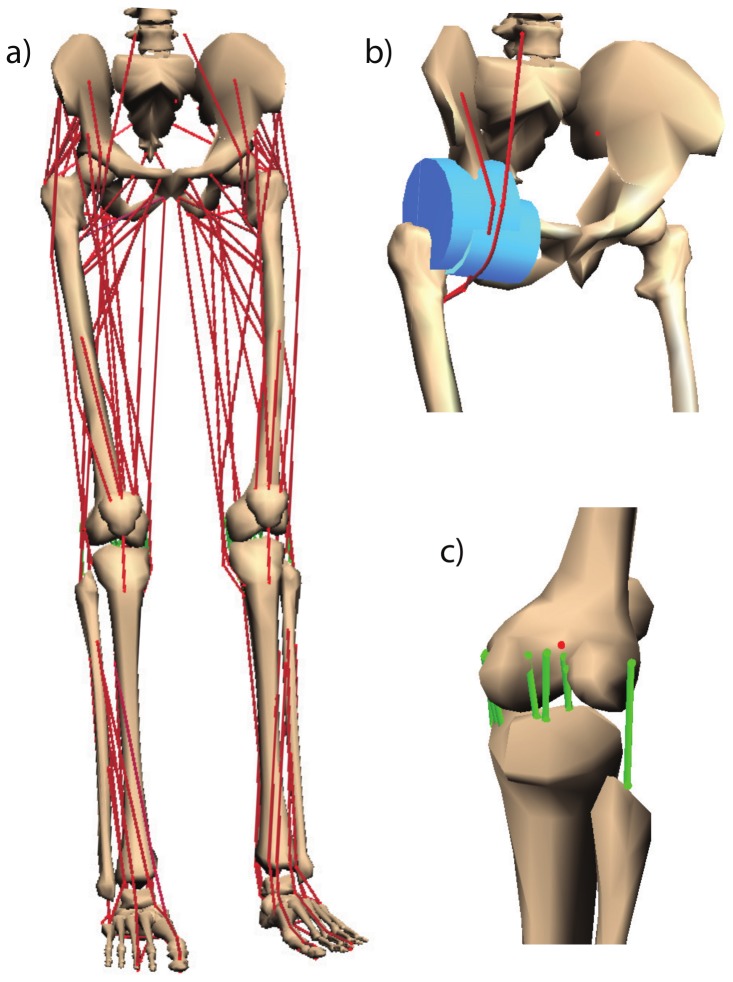
Musculoskeletal model of the lower limb. (**a**) Full model with the 44 Hill-type muscle tendon units. (**b**) Example of two wrapping objects to constrain the muscle–tendon path. (**c**) Ligaments of the right knee.

The muscle–tendon parameters used in our simulator were reported in [30]. Here, a post-mortem analysis was performed to 21 cadavers with a weight and height of 82.7 ± 15.2 kg and 168.4 ± 9.3 cm, respectively. The musculoskeletal model is depicted in Figure 6, and Table 2 shows the morphometric parameters.

**Figure 7:**
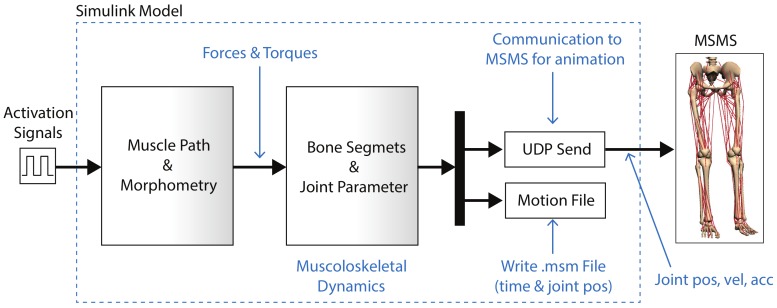
Simulink block model and MSMS integration.

After that, the biomechanical model was exported to Simulink to merge simulated data with external data measured by IMUs and EMG sensors if needed. The Simulink model was composed by a drivers block with the muscle–tendons features (morphometry, fiber type, and muscle path) in which the muscle signals were set, a block representing the bone segments (SimMechanics block), an UDP block to send the signals to MSMS, and a block that generated a file (“.msm”) used by MSMS as a source animation file. Figure 7 shows the Simulink equivalent block diagram and its relation with MSMS.

**Figure 8:**
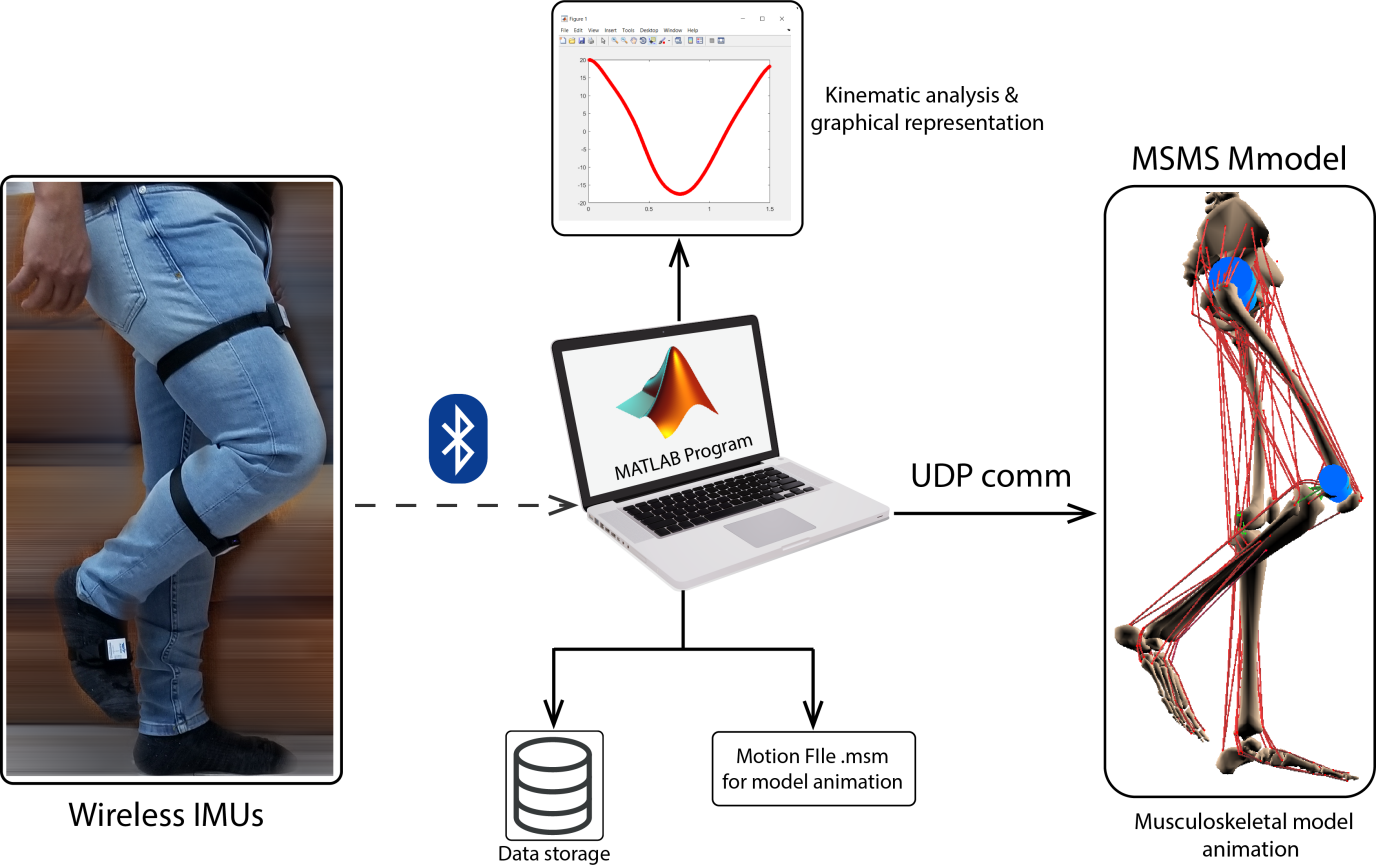
Wireless gait caption system [32].

The patient gait was registered and then included in the control loop (Figure 8). The inertial sensors used were wireless with nine axes: each module consisted of a high precision three axis gyroscope, three axis accelerometer, three axis geomagnetic sensor, and a 32 bit high performance MCU. According to the manufacturer, the IMU allowed us to have an output rate up to 200 Hz and an accuracy of 0.05${}^{\circ }$.

## 5. ALICE Control Architecture

The literature reports several control strategies for lower limb robotics exoskeletons, from the simplest PD to advanced controllers like the sliding adaptive controllers [14,33,34,35,36,37]. One of the most important problems to solve is approximating the tracking error to zero when a torque disturbance is found in the system. In the case of gait rehabilitation, each patient, disease, or treatment is different; then, the control strategy must be automatic or easy to adapt to each situation. ALICE is a unique robotic exoskeleton that uses the on-board musculoskeletal model to adapt the control signal automatically to the patient’s particular condition. An adaptive PD controller was designed using internal and external sensors (for the robot and patient) and data computed from the models.

### Adaptive-PD Controller

**Figure 9:**
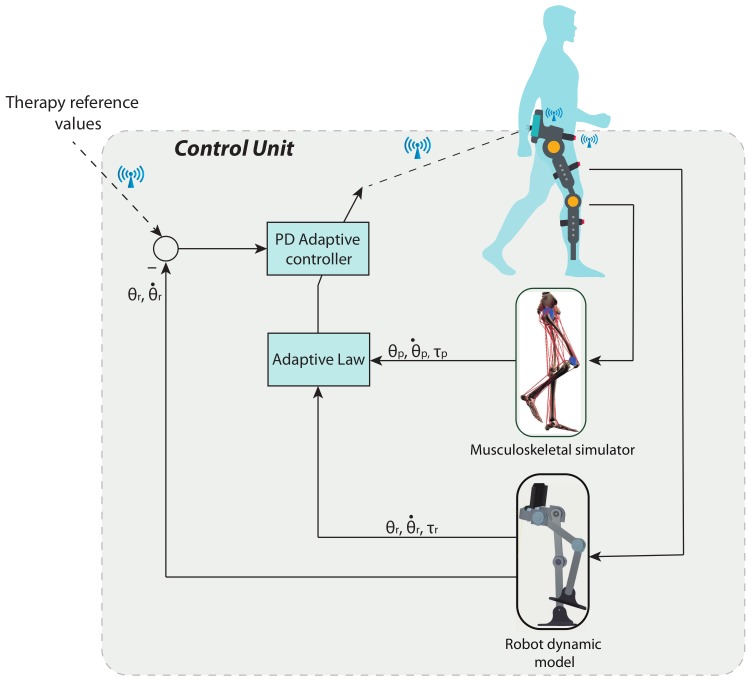
Control concept, adaptive PDcontroller.

Figure 9 presents the control law used in this project. In this section, we will design the controller using Lyapunov’s theory.

In this controller, the kinetics of patient’s movements was considered as a disturbance input torque, then it was added to (25),

(27) $$D_{n}\ddot{q}+C_{n}\dot{q}=\tau_{\Phi n}+\tau_{dn}$$

where $\tau_{dn}$ is the disturbance torque. Multiplying $D_{n}^{-1}$ on both sides of (27), we obtain:

(28) $$\ddot{q}+D_{n}^{-1}C_{n}\dot{q}=D_{n}^{-1}\tau_{\Phi n}+D_{n}^{-1}\tau_{dn}$$

considering the virtual input and disturbance torques as $\tau_{qn}=D_{n}^{-1}\tau_{\Phi n}$ and $\tau_{qd}=D_{n}^{-1}\tau_{dn}$, respectively.

In order to design the adaptive PD controller, it is necessary to convert (28) to state variables by means of the variables $z_{1}=q$ and $z_{2}=\dot{q}$, that is:

(29) $$\begin{array}{ccc} {\dot{z}}_{1} & = & z_{2}\\ {\dot{z}}_{2} & = & \tau_{qn}+\tau_{qd}-D_{n}^{-1}C_{n}z_{2} \end{array}$$

Thus, the PD adaptive controller is given by:

(30) $$\tau_{qn}=K_{1}e_{1}+K_{2}e_{2}$$

where $e_{1}=q_{r}-q=z_{1r}-z_{1}$ and $e_{2}={\dot{q}}_{r}-\dot{q}=z_{2r}-z_{2}$. Moreover, $q_{r}=z_{1r}$ is the reference vector, ${\dot{q}}_{r}=z_{2r}$ the reference vector derivative, and $K_{1}$ and $K_{2}$ the proportional and derivative gain, respectively.

In order to obtain the adaptive control laws and ensure that the errors $e_{1}\to 0$ and $e_{2}\to 0$ when the time approaches infinity, it is necessary to define the following dynamics of the error,

(31) $$\begin{array}{ccc} {\dot{e}}_{1} & = & {\dot{z}}_{1r}-{\dot{z}}_{1}={\dot{z}}_{1r}-z_{2}\\ {\dot{e}}_{2} & = & {\dot{z}}_{2r}-{\dot{z}}_{2}={\dot{z}}_{2r}-\tau_{qn}-\tau_{qd}+D_{n}^{-1}C_{n}z_{2} \end{array}$$

substituting $\tau_{qn}$ into (31), we obtain:

(32) $$\begin{array}{ccc} {\dot{e}}_{1} & = & {\dot{z}}_{1r}-z_{2}\\ {\dot{e}}_{2} & = & {\dot{z}}_{2r}-K_{1}e_{1}-K_{2}e_{2}-\tau_{qd}+D_{n}^{-1}C_{n}z_{2} \end{array}$$

The following property is necessary in order to obtain the adaptive law by the Lyapunov method, considering that the disturbance torque norm $\tau_{qd}$ has an upper limit.

**Property** **1.** *The disturbance torque norm $\tau_{qd}$ has the following upper limit:*

(33) $$∥\tau_{qd}∥<\delta$$

*for δ>0.*

Moreover, to find the adaptive control law, it is necessary to define the following Lyapunov function:

(34) $$V=\frac{1}{2}e_{1}^{T}e_{1}+\frac{1}{2}e_{2}^{T}e_{2}+\frac{1}{2}K_{1}^{2}+\frac{1}{2}K_{2}^{2}$$

taking the derivative of Equation (34), we obtain:

(35) $$\begin{array}{ccc} \dot{V} & = & e_{1}^{T}\left[{\dot{z}}_{1r}-z_{2}\right]\\ & + & e_{2}^{T}\left[{\dot{z}}_{2r}-K_{1}e_{1}-K_{2}e_{2}-\tau_{qd}+D_{n}^{-1}C_{n}z_{2}\right]\\ & + & K_{1}{\dot{K}}_{1}+K_{2}{\dot{K}}_{2} \end{array}$$

Substituting the following adaptive law in (35):

(36) $$\begin{array}{ccc} {\dot{K}}_{1} & = & \frac{-1}{K_{1}}\left[K_{1}e_{1}^{T}{\dot{z}}_{1r}-K_{1}e_{1}^{T}z_{2}-e_{2}^{T}K_{1}^{2}e_{1}\right]\\ {\dot{K}}_{2} & = & \frac{-1}{K_{2}}\left[K_{2}e_{2}^{T}z_{2r}-e_{2}^{T}K_{2}^{2}e_{2}+K_{2}e_{2}^{T}D_{n}^{-1}C_{n}z_{2}\right] \end{array}$$

yields,

(37) $$\dot{V}=-e_{2}^{T}\tau_{qd}$$

Thus, applying the norm to both sides of (37) and implementing the Property 1, we obtain,

(38) $$\dot{V}\leq -\delta ∥e_{2}^{T}∥$$

From this result, it is proven that adaptive laws (36) ensure the closed loop stability of the exoskeleton for the respective trajectory tracking.

## 6. Simulation Results with Real Data

The design process of a robotics rehabilitation system is complex, and testing it with patients in a real clinical environment requires the approval of legal authorities and accomplishing at least safe regulatory issues. This proposal is in TRL 4–5. and it is far from real testing in the clinical environment. However, it is necessary to validate each new step of the development with the greatest possible level of reliability. Therefore, for the simulation results, we used real gait data captured with commercial medical equipment, CODA^TM^ Motion Analysis System, from a patient with multiple sclerosis at the Physiotherapy Clinic of the ONCE Foundation.

**Figure 10:**
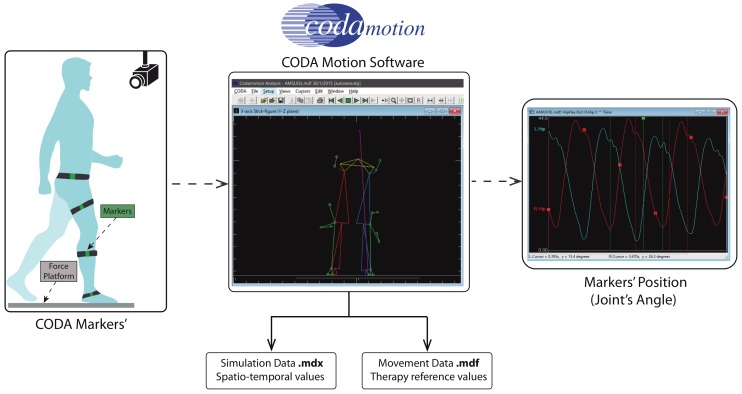
Simulation scheme in CODA motion software to obtain therapy reference values.

Figure 10 shows the simulation scheme to obtain the therapy reference values. These trajectories were sent as reference signals to the control system. Data used in the simulation of proposed control law were collected from four patients diagnosed with multiple sclerosis, which is a neuro-degenerative disease with a high variability among patients, and a healthy volunteer. All subjects were in the same range of age, height, and weight, as shown in Table 3.

Most relevant mechanical parameters of the robot such as length and mass parameters are defined in Table 4, while inertia matrices are shown in Equation (39). The values of ALICE’s links were determined from the anthropometric data shown in [38,39].

**Figure 11:**
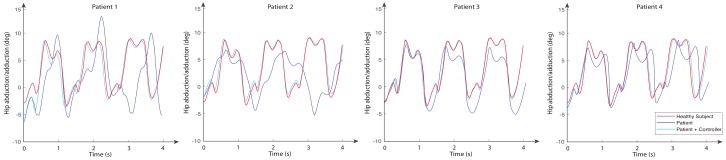
Hip abduction/adduction angles for a normal subject (reference signal) and four subjects with multiple sclerosis for both open loop and using the PD adaptive controller.

**Figure 12:**
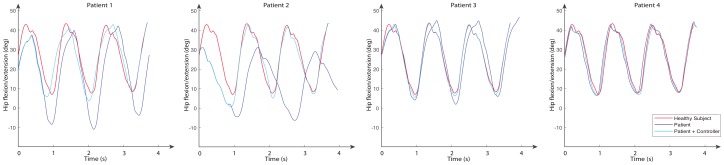
Hip flexion/extension angles for a normal subject (reference signal) and four subjects with multiple sclerosis for both open loop and using the PD adaptive controller.

**Figure 13:**
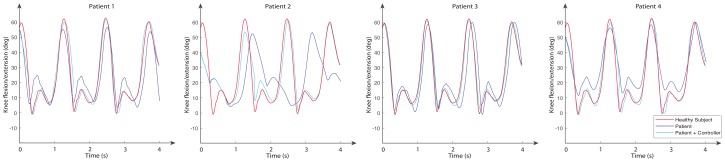
Knee flexion/extension angles for a normal subject (reference signal) and four subjects with multiple sclerosis for both open loop and using the PD adaptive controller.

The movements of hip abduction/adduction, hip flexion/extension, and knee flexion/extension angles for these subjects are shown in Figure 11, Figure 12 and Figure 13, respectively. The figures also include the gait pattern after applying the PD adaptive controller. In this simulation, we assumed all patients had the same references values; however, in a real rehabilitation process, reference values are established by medical staff taking into account the particular condition of their patient.

(39) $$\begin{array}{c} I_{1}=\left[\begin{array}{ccc} 0.004977351 & 0 & 0\\ 0 & 0.004835199 & 0\\ 0 & 0 & 0.001191692 \end{array}\right]\\ I_{2}=\left[\begin{array}{ccc} 0.009337758 & 0 & 0\\ 0 & 0.166430481 & 0\\ 0 & 0 & 0.169652773 \end{array}\right]\\ I_{3}=\left[\begin{array}{ccc} 0.019400497 & 0 & 0\\ 0 & 0.135966856 & 0\\ 0 & 0 & 0.146655675 \end{array}\right] \end{array}$$

The numerical simulation showed how the tracking error of each joint approached zero as time approximated two seconds. It is important to mention that the adaptive PD controller, in opposition to other approaches found in the literature, provided a robust control approach in the presence of external disturbances. These external disturbances were generated by the gait of an unhealthy patient, which affected the torque yielded by the actuators. These kinds of disturbances of courses are considered bounded because the unhealthy patient disturbance trajectory is known a priori. This condition is established theoretically, and the PD adaptive laws are designed by an appropriate Lyapunov approach. It can be noticed in Figure 11, Figure 12 and Figure 13 that the trajectory was tracked accurately by implementing the proposed controller despite the external disturbance yielded by an unhealthy patient. In opposition, as a standard PD controller, the adjustment of the PD controller gains improved the closed loop performance even when disturbance could drive the system variables, which in this case were the actuator joints, to instability in unexpected conditions.

## 7. Discussion

The rehabilitation services either in public or private health systems are overloaded due to the effect of population aging and the rise in incidence of strokes, especially in women. In such cases, restoring the patient to his/her normal life would depend, among other factors, on the quality of rehabilitation: onset, duration, and frequency of therapy. Rehabilitation robots arise as a potential solution to solve some of these daily problems in the rehabilitation services.

Certainly, many efforts have been made in the research and development of rehabilitation robots, yet the solution continues to be far from being used in clinical practices. As we mentioned before, the economic cost of the technology constitutes an important barrier to its massive scale-up.

In this article, we presented the ALICE project. The system is a mechatronics development leading to assisting rehabilitation staff in the entire procedure of the diagnosis and rehabilitation of the pathological gait. ALICE is able to give information about both the kinematics and kinetics of the patient’s movements in real time.

The kinematic information is available in almost all commercial and research exoskeletons and is used to control the robot during the gait cycle. The kinematics of lower limb is widely studied and described in the literature [40,41,42,43].

In ALICE, we used a hybrid approach by combing both data measured by sensors and data computed by models. Moreover, we assumed that the robot was attached to the patient’s lower limb where inertial measurement units (IMUs) were localized. Furthermore, in this paper, we describe the kinematic model of the designed robotic mechanism. The model was developed under the unit quaternion theory, which is one of the most powerful tools for computing in real time.

Regarding kinetic data, they are not easy to obtain. Each person has his/her own inherent pattern for walking, using different postural, neuromuscular, locomotor, and dynamic balance control. Some studies [44,45,46] showed there were different patterns for healthy people and many others differed under some pathological condition. Moreover, these patterns depended on external parameters such as velocity and contact surfaces.

ALICE project advances the state-of-the-art in kinetic computing of the human gait by using an on-board musculoskeletal simulator to obtain the forces of internal muscles. Despite the forces of external muscle being easily measured by non-invasive electromyography technology (EMG), the internal muscle forces could be measured only by an invasive technique called Needle EMG. It potentially produces iatrogenic effects such as bleeding (a little bleeding is the rule), hematoma, infection, and pneumothorax [47,48]. Our proposal brings out a full lower limb musculoskeletal model running on-board and computing the internal forces of the movement without invasion. The reader is referred to [15] for details.

An adaptive PD controller was designed to close the loop. We chose this control architecture because once the stable locomotion is ensured, the controller parameters take into consideration the personal condition of the patient. According to the obtained results, it was observed that the adaptive PD controller provided a very good dynamic response, generating an accurate track of the trajectory for patients with multiple sclerosis. In addition, it can be seen that the actuators $q_{1}=\theta_{1}$ and $q_{3}=\theta_{3}$ reduced the error when the time approached 1 s. In the case of the actuator $q_{2}=\theta_{2}$, the error was reduced when the time approached 2 s, which verified the effectiveness of the adaptive PD controller.

In addition, it is important to point out that while the results obtained from this experiment were acceptable, these could be improved in the future by testing them with a sliding mode controller or a robust controller.

### Robotics Devices in the New European Regulatory Context

In this article, a conceptual design of a complex sensor to measure/estimate human gait parameters is presented, including a lower limb robot that can also assist in the gait cycle. ALICE aims to be a medical device suitable for clinical practice, then its validation must be carried out in that environment, with a specific medical protocol. A validation in a different environment would not be a realistic validation of the device.

Until now, the authorization of hospital ethical committees was enough to perform a first clinical validation in order to improve and test the device. However, in the new European regulatory framework (deadline May 2020), it is required for any clinical validation involving humans to be explicitly authorized by the government’s medical agencies.

This validation already requires a quasi-commercial product, for example: user manual, risk analysis, pre-manufacturing process, payment of insurance for each enrolled patient, pre-clinical trial approved (certification for electrical and electromagnetic compatibility, RoHS, machine tool regulatory, etc). Therefore, reducing the gap between lab and clinical practice will be more difficult than before.

## 8. Conclusions

In this article, a novel concept for a robotic exoskeleton was described. The main innovation of our project lied in the kinetic and kinematic data from both the robot and patient, which were included in the control loop, making ALICE a novel sensor useful for diagnosis, rehabilitation, and objective quantification of the therapy progress.

Using Hamilton quaternions, a dynamic analysis was performed through the Euler–Lagrange formulation and expressed in state variables. Furthermore, a full musculoskeletal model was presented including 14 bone segments, 88 muscle–tendon units, 20 knee ligaments, and 12 joints, which allowed simulating the kinetic data of the patient.

An adaptive PD controller architecture was evaluated in order to personalize the therapy to each patient and to improve his/her gait as much as possible. The test data were taken from our own wireless gait capture system for healthy volunteers and patients diagnosed with multiple sclerosis disease. All information, sensor data, and information provided by the models are available for medical staff.

The next step in our development is to reach TRL 6, where an exoskeleton will be prototyped using the power and control electronics, as well as electrical actuators selected in the analysis presented is this paper. This advance in TRL will allow validating the hardware component, ergonomic aspects, control strategies, and measurement capabilities of the proposed platform. 

## Figures and Tables

**Table 1:** Joints and Range of Motion (ROM).

Joint	Action	ROM
	Extension/Flexion	$-30^{\circ }/120^{\circ }$
Hip	Abduction/Adduction	$-50^{\circ }/30^{\circ }$
Knee	Flexion/Extension	$-120^{\circ }/0^{\circ }$
Ankle	Plantar/Dorsal Flexion	$-40^{\circ }/30^{\circ }$

**Table 2:** Morphometrics values of the model [15].

Muscle Segment	Optimal Fascicle Length (cm)	Optimal Tendon Length (cm)	Max. MT Length (cm)	Mass (g)	PCSA(cm${}^{2}$)	Max. Force (N)
Adductor brevis	10.30	3.60	16.68	104.3715	9.55958	303.994
Adductor longus	10.82	13.00	28.58	144.0863	12.5628	399.499
Adductor mag.distal	17.72	9.00	32.06	191.49	10.1949	324.2
Adductor mag. ischial	15.62	22.1	45.26	168.8	10.1949	324.2
Adductor mag. middle	13.77	4.8	22.8	148.8078	10.1949	324.2
Adductor mag. proximal	10.56	4.3	17.83	114.1184	10.1949	324.2
Biceps femoris long head	9.76	32.2	50.35	229.425	22.1761	705.2
Biceps femoris short head	11.03	10.4	25.72	116.1091	9.9308	315.8
Extensor digitorum long.	6.93	36.73	52.39	79.7874	10.8616	345.4
Extensor hallucis longus	7.48	33.15	48.76	41.14	5.1886	165
Flexor digitorum longus	4.46	37.77	50.68	40.794	8.6289	2274.4
Flexor hallucis longus	5.27	35.6	49.04	76.7312	13.7358	436.8
Gastrocnemius lateral	5.88	38.2	52.90	118.854	19.069	606.4
Gastrocnemius medial	5.1	40.08	54.22	222.359	41.132	1308
Gemelli	2.4	3.9	7.56	8.72	3.4276	109
Gluteus max. superior	14.73	5.0	23.68	268.135	17.1729	546.1
Gluteus maximus middle	15.69	7.33	27.62	408.2015	24.544	780.5
Gluteus maximus inferior	16.65	7.02	28.40	291.9855	16.544	526.1
Gluteus medius anterior	7.33	5.65	15.58	215.2821	27.707	881.099
Gluteus medius middle	7.33	6.6	16.72	150.6315	19.3867	616.5
Gluteus medius posterior	7.33	4.6	14.32	171.522	22.075	702
Gluteus min.anterior	6.8	1.6	10.08	40.8	5.6603	180
Gluteus min. middle	5.6	2.6	9.84	35.466	5.9748	190
Gluteus min. posterior	3.8	5.1	10.68	27.23	6.761	215
Gracilis	22.78	16.6	47.26	104.256	4.3176	137.3
Iliacus	10.66	9.4	24.07	220.98	19.556	621.9
Patellar tendon	5.0	0.5	6.6	0.1666	0.03144	1.0
Pectineus	13.3	0.1	16.08	78.47	5.566	177
Peroneus brevis	4.54	14.81	23.22	46.2928	9.619	305.9
Peroneus longus	5.08	33.3	46.06	110.6254	20.544	653.299
Peroneus tertius	7.9	10.0	21.48	23.7	2.83	90
Piriformis	2.6	11.5	16.92	25.6533	9.308	296
Psoas	11.69	9.7	25.67	186.923	15.0849	479.7
Quadratus femoris	5.4	2.4	9.36	45.72	7.9874	253.99
Rectus femoris	7.59	34.6	50.63	214.746	26.691	848.8
Sartorius	40.3	11.0	61.56	152.468	3.569	113.5
Semimembranosus	6.9	34.8	50.04	267.421	36.562	1162.7
Semitendinosus	19.3	24.5	52.56	194.2	9.4937	301.9
Soleus	4.4	28.15	39.06	525.93	112.76	3585.899
Tensor fascia latae	9.5	45	65.4	49.083	4.874	155
Tibialis anterior	6.83	24.1	37.12	153.379	21.1855	673.7
Tibialis posterior	3.78	28.18	38.35	114.1055	28.4779	905.599
Vastus intermedius	9.93	10.6	24.64	339.0102	32.2075	1024.2
Vastus lateralis	9.94	13.0	27.53	747.289	70.9245	2255.4
Vastus medialis	9.68	11.2	25.06	465.833	45.399	1443.7

**Table 3:** Patients’ anthropometric data.

Patient	Age	Height (cm)	Weight (kg)	Pelvic Width (cm)	Femur Length (cm)	Tibia Length (cm)
1	49	1.57	60	34	37	36
2	47	1.63	58	33	39	37
3	52	1.68	68	34	40	39
4	47	1.62	62	35	39	38

**Table 4:** ALICE length and mass values.

Parameter	Value
$l_{1}$	0.115 m
$l_{2}$	0.160 m
$l_{3}$	0.400 m
$l_{4}$	0.400 m
$m_{1}$	1.18 kg
$m_{2}$	13.228 kg
$m_{3}$	5.594 kg
